# Energy Drink Knowledge, Consumption, and Regulation Support Among Polish Medical and Non-Medical Students: A Cross-Sectional Study

**DOI:** 10.3390/nu17213430

**Published:** 2025-10-31

**Authors:** Paulina Mularczyk-Tomczewska, Tytus Koweszko, Julia Koperdowska, Ewelina Adamska, Andrzej Silczuk

**Affiliations:** 1 Department of Public Health, Faculty of Health Sciences, Medical University of Warsaw, 02-091 Warsaw, Poland; 2 Department of Community Psychiatry, Faculty of Health Sciences, Medical University of Warsaw, 02-091 Warsaw, Poland; tytus.koweszko@wum.edu.pl (T.K.); andrzej.silczuk@wum.edu.pl (A.S.); 3 Faculty of Health Sciences, Medical University of Warsaw, 02-091 Warsaw, Poland; juliakoperdowska@gmail.com (J.K.); s086927@student.wum.edu.pl (E.A.)

**Keywords:** energy drinks, caffeine, students, risk perception, sociodemographic factors, public health policy

## Abstract

**Background:** Energy drink [ED] consumption is common among young adults and has been linked to adverse health effects and risky behaviors. This study compared medical and non-medical university students to assess whether health education influences knowledge, consumption, and attitudes toward EDs. Although medical and non-medical students are not minors, their opinions on the national ban on EDs sales to individuals under 18 provide valuable insight into attitudes toward regulation. **Material and Methods:** A cross-sectional online survey was conducted among 871 students (42.1% medical, 57.9% non-medical). The questionnaire assessed demographics, ED consumption, knowledge, motivations, and regulatory attitudes. It was pilot-tested on 30 students to ensure clarity, and internal consistency was confirmed (Cronbach’s α = 0.78 for knowledge; α = 0.81 for attitudes). Non-parametric tests (Mann–Whitney U, Kruskal–Wallis) and chi-square analyses compared groups. **Results:** Participants’ mean age was 22.1 years; most were female (73.2%). Medical students demonstrated significantly better knowledge of ED ingredients (simple sugars, B vitamins, L-carnitine, electrolytes; *p* < 0.01) and adverse effects (e.g., irritability, dizziness, nausea; *p* < 0.05). However, ED consumption frequency did not differ between medical and non-medical students. The main reasons for ED use were energy and concentration; social motives were less frequent. Female students more often supported the ban on ED sales to minors and additional advertising restrictions (*p* < 0.001), while overall confidence in enforcement was low. **Conclusions:** Despite greater awareness, medical students consume EDs at rates comparable to non-medical students. Educating medical students on safe caffeine use is crucial, since shift work may promote stimulant intake. Combining targeted education with stronger enforcement could enhance the impact of regulatory policies and reduce risky consumption among young adults.

## 1. Introduction

Over the past three decades, energy drinks [EDs] have become one of the fastest growing segments of the global beverage market. Their wide availability and intensive marketing have made them particularly popular among adolescents and young adults, who now represent the largest consumer group [1,2,3,4]. EDs are increasingly recognized as a public health concern due to their growing consumption among young people worldwide [5].

EDs are widely consumed by young people. Their use often begins in adolescence and is more prevalent among males and physically active individuals. It is frequently associated with behaviors such as smoking and alcohol use, as well as adverse health effects including palpitations, whereas marketing campaigns tend to emphasize supposed improvements in cognition and performance benefits while underestimating associated risks [6,7,8].

ED consumption is associated with lifestyle factors and irregular dietary behaviors, with sociodemographic characteristics including male gender, unmarried status, and enrollment in non-medical fields of study [9,10,11]. Students facing intensive academic demands may also rely on EDs to cope with fatigue and maintain alertness, despite their association with adverse health outcomes including sleep problems, migraines, and cardiovascular complications [12]. Among university students, knowledge of these products is often limited, attitudes remain ambivalent, and reported motives for use include prolonging wakefulness, enhancing study capacity, and increasing energy levels [13].

Medical students may differ from non-medical students in both their knowledge of ED ingredients and their attitudes toward potential health risks, indicating that the field of study could influence patterns of use and perceptions [11,12,13]. Although EDs are often consumed by students to improve concentration and academic performance, the scientific evidence for such benefits is weak, and growing concerns highlight their potential negative consequences for mental health and overall well-being, reinforcing the need for further research [6,14]. Research among university students has shown that ED use is shaped not only by individual motives and perceived benefits but also by social influences, misinformation about product content, and the easy accessibility of these beverages [15]. High rates of ED use have also been observed among medical students, with study-related demands and the need for energy as the main motives, and mass media and peers serving as key sources of information [16].

Recent evidence from Poland shows that while public awareness of the health risks associated with EDs is relatively high and support for age-related sales restrictions is strong, many individuals doubt the effectiveness of such measures in limiting access among minors [4]. In response to these concerns, Poland has introduced nationwide regulations governing the sale and distribution of EDs. A key step was the 2023 amendment to the Public Health Act, which came into force on 1 January 2024. Under this law, the sale of EDs to individuals under 18 years of age is prohibited, and their distribution is banned in schools as well as through vending machines. Retailers are authorized to request proof of age when necessary. The Act also provides an official definition of an “energy drink” and requires clear product labeling, aiming to support consumer awareness and facilitate enforcement. However, despite this legal framework, questions remain regarding the effectiveness of enforcement measures and the actual availability of EDs to minors [3,17].

The study aimed to assess ED consumption and related attitudes among students in the Mazovia region of Poland. Specifically, the objectives were to examine (1) the frequency and motives of ED use, (2) students’ knowledge of their health effects, and (3) opinions regarding the recently introduced sales ban and the potential need for additional restrictions, with a particular focus on differences between medical and non-medical students.

## 2. Materials and Methods

### 2.1. Questionnaire and Study Measures

This cross-sectional survey was conducted among university students, including both medical and non-medical faculties, in the Mazovia region of Poland between 1 and 20 July 2025. Medical students were selected as a key group because, as future healthcare professionals, they are exposed to high academic demands, stress, and irregular schedules that may predispose them to stimulant use. Data were collected using an anonymous online questionnaire distributed via multiple social media platforms, including student forums and university groups. Participation in the study was voluntary and preceded by informed consent obtained electronically after participants read a statement outlining the study aims, anonymity, and their right to withdraw at any time. Eligibility criteria included being enrolled as a student at a higher education institution located in Mazovia at the time of the study. The questionnaire included sections on sociodemographic characteristics, consumption patterns of ED, knowledge of their composition and potential adverse effects, and attitudes toward legal regulations concerning these products. The study protocol was reviewed and approved by the Bioethics Committee at the Medical University of Warsaw, Poland (decision number AKBE/139/2025).

The study used an author-developed questionnaire designed based on a review of previous surveys investigating energy drink consumption, motivations for use, and regulatory attitudes [4,5,9,11,13]. Although informed by prior research, the instrument was original and tailored to the Polish context, including new items addressing the awareness of the recent national ban on energy drink sales to minors. It comprised sections on sociodemographic characteristics, knowledge, consumption behaviors, and attitudes toward legal regulations concerning EDs. Content validity was verified by three academic experts in public health prior to pilot testing. Prior to data collection, the questionnaire was pilot-tested on 30 students not included in the final sample to assess clarity and completion time. Minor modifications were made to improve comprehensibility. Internal consistency of the main multi-item sections was assessed using Cronbach’s alpha, which showed satisfactory reliability (knowledge α = 0.78; attitudes α = 0.81). Knowledge of ED was assessed through questions regarding awareness of specific ingredients (e.g., caffeine, taurine, guarana, simple sugars, B vitamins, L-carnitine, electrolytes) and potential adverse health effects (e.g., irritability, nausea, dizziness, sleep problems, increased blood pressure, impaired coordination). Consumption behaviors were measured by asking whether respondents had ever consumed EDs, the age of initiation, frequency of consumption, and whether they combined EDs with other substances such as alcohol, coffee, cigarettes, e-cigarettes, dietary supplements, or psychoactive substances. Additional questions concerned the reasons for consumption, including motivation related to studying, concentration, energy boost, leisure activities, or social situations. Attitudes toward legal regulations were assessed using a 5-point Likert scale (1 = definitely not, 5 = definitely yes). Respondents were asked whether they supported the national ban on the sale of EDs to individuals under 18, whether they perceived the ban as effective in reducing availability, and whether they supported additional restrictions such as advertising limitations. For some analyses, answers “definitely yes” and “mostly yes” were combined to indicate agreement. Sociodemographic data included gender (female, male, other, prefer not to answer), age (years), place of residence (rural area, town ≤ 20,000 residents, city 20,000–99,999 residents, city 100,000–499,999 residents, city ≥ 500,000 residents), type and year of study program (bachelor, master, integrated master’s), mode of study (full-time, part-time), and self-assessed financial situation (very good, good, moderate, poor, very poor). The selected sociodemographic variables are commonly included in studies examining energy drink consumption and related behaviors, which allows for meaningful comparison with previous findings.

### 2.2. Sample Size and Recruitment

Recruitment followed a convenience sampling approach, with open access to the online survey link. Although non-random sampling may lead to self-selection bias, efforts were made to minimize this risk by disseminating the survey widely across various student groups representing different fields of study. A minimum required sample size was estimated using G*Power (version 3.1.9.7) for the chi-square test (medium effect size, α = 0.05, power = 0.80), indicating a threshold of 384 participants [18]. The final sample of 871 students substantially exceeded this number, ensuring adequate statistical power.

### 2.3. Statistical Analysis

All analyses were performed using IBM SPSS Statistics, version 29 (IBM Corp., Armonk, NY, USA). Normality of distributions was assessed using the Shapiro–Wilk test, which indicated the need for non-parametric methods for several variables. Descriptive statistics were calculated for all variables and presented as means with standard deviations (SDs) for continuous variables and as frequencies with percentages for categorical variables. To examine differences between medical and non-medical students, as well as across sociodemographic groups, non-parametric tests were applied. The Mann–Whitney U test was used to compare two independent groups (e.g., male vs. female students), while the Kruskal–Wallis test with post hoc pairwise comparisons was applied for comparisons across more than two groups (e.g., place of residence). The median test was additionally employed to assess differences in central tendency. Associations between categorical variables were examined using chi-square (χ^2^) tests of independence. Binary logistic regression models were applied to identify sociodemographic predictors of knowledge, consumption, and attitudes toward ED regulations. The enter method was used, and multicollinearity was checked with variance inflation factors (VIF < 2). Results are presented as odds ratios (ORs) with 95% confidence intervals (CIs). For all analyses, a *p*-value of less than 0.05 was considered statistically significant.

## 3. Results

### 3.1. Characteristics of the Study Group

The study sample consisted of 871 university students, including 367 (42.1%) medical students and 504 (57.9%) non-medical students. The mean age was 22.1 years (SD = 3.05; range: 18–45). Most respondents were female (73.2%, *n* = 638), while 25.3% (*n* = 220) identified as male; 0.8% (*n* = 7) reported “other,” and 0.7% (*n* = 6) preferred not to disclose their gender. The majority of participants resided in very large cities (>500,000 inhabitants, 66.8%). Smaller shares lived in rural areas (10.2%), small towns of up to 20,000 inhabitants (5.4%), medium-sized cities of 20,000–100,000 (10.9%), and large cities of 100,000–500,000 (6.7%). In terms of financial situation, 28.7% described their household status as very good, 59.4% as good, 9.9% as moderate, and 2.1% as poor or very poor. Regarding education, 37.7% of students were pursuing bachelor’s studies, 21.2% master’s studies, and 41.1% integrated master’s programs. Most were full-time students (85.3%), with the remainder studying part-time (14.7%). Distribution by year of study was as follows: first year 23.1%, second year 26.1%, third year 23.0%, fourth year 15.6%, fifth year 9.4%, and sixth year 2.9%. Detailed sociodemographic characteristics of the study population, including age, gender, field of study, and place of residence, are presented in Table 1.

### 3.2. Frequency and Patterns of Energy Drinks Consumption

Overall, 75.2% of university students reported consuming EDs at least occasionally, while 24.8% indicated that they never consumed them. The most common frequencies of consumption were 2–3 times per week 14.2%, once per month 13.9%, and less than once every three months 14.5%. Daily or almost daily intake was reported by 9.2% of respondents. When comparing fields of study, 75.8% of medical students and 73.2% of non-medical students reported ED use. This difference was not statistically significant. The frequency of ED consumption did not differ significantly between medical and non-medical students. Gender-based comparisons showed that male students reported significantly more frequent ED consumption than females (*p* < 0.05).

Similarly, no significant differences were observed in the co-consumption of EDs with alcohol, cigarettes, e-cigarettes, dietary supplements, or psychoactive substances. However, medical students reported combining EDs with coffee more frequently than non-medical students (16.6% vs. 10.7%; χ^2^ = 6.47; *p* = 0.011). Most students reported first trying energy drinks between the ages of 15 and 18 years (37.7%), while nearly one-third (29.8%) consumed them before the age of 15. Among medical students, 41.4% first consumed energy drinks between ages 15–18 and 42.0% before 15, compared with 58.6% and 58.0% among non-medical students, respectively. Non-medical students thus tended to start slightly earlier, though the difference was not statistically significant.

### 3.3. Motivations for Consumption of Energy Drinks

The most common motivations for energy drink (ED) consumption were increased energy (63.1%), improved concentration while studying (37.3%), and taste (37.4%), followed by using EDs as a coffee substitute (32.0%). Statistical analysis (Mann–Whitney U test with continuity correction) revealed that medical students more frequently reported energy enhancement (Z = 2.48; *p* = 0.013) and improved concentration (Z = 4.69; *p* < 0.001) as reasons for ED use compared with non-medical students. No significant differences were found for leisure or socially driven motives, such as peer pressure.

### 3.4. Knowledge About Energy Drinks

When asked about the safe daily dose of caffeine for healthy adults, only 31.3% of students correctly identified the recommended limit of 400 mg. A further 25.4% indicated 200 mg, and 8.1% selected 100 mg, while 35.3% declared that they did not know the correct answer. Regarding caffeine content, medical students significantly more often selected the correct value referring to the maximum recommended daily intake of caffeine for adults (400 mg) compared to non-medical students (*p* < 0.001).

Comparisons between medical and non-medical students revealed significant differences in knowledge of certain ED ingredients. Medical students were more likely to correctly identify simple sugars, B vitamins, L-carnitine, and electrolytes (*p* < 0.01). No significant differences were observed for caffeine, taurine, guarana, ginseng, or artificial colorants.

**Figure 1:**
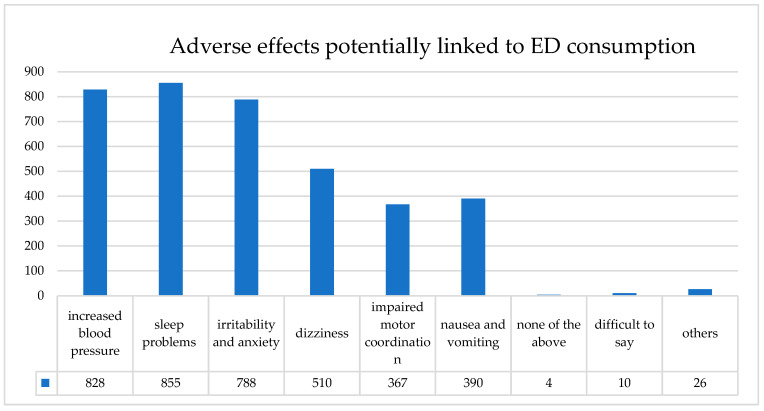
Self-reported adverse effects potentially associated with ED consumption among university students (*n* = 871). The most frequently indicated effects were sleep problems (98.2%), increased blood pressure (95.1%), and irritability and anxiety (90.4%) (multiple responses possible).

Moreover, medical students demonstrated greater awareness of adverse effects of ED consumption, including irritability, dizziness, nausea, and impaired coordination (*p* < 0.05). The most frequently reported adverse effects among all respondents were sleep problems (98.2%), increased blood pressure (95.1%), and irritability and anxiety (90.4%), followed by dizziness (58.5%), nausea and vomiting (44.8%), and impaired motor coordination (42.1%) (Figure 1). No significant differences were noted for increased blood pressure or sleep problems.

### 3.5. Attitudes Toward Energy Drink Regulations and Sociodemographic Differences

Support for the legal ban on the sale of EDs to minors was high in both study groups. Gender was significantly associated with regulatory attitudes: female students were more likely than male students to support the ban (U = 53,143.5; Z = 6.09; *p* < 0.001) and to endorse additional restrictions such as advertising limitations (U = 47,437.5; Z = 7.40; *p* < 0.001). However, no significant gender differences were observed in perceptions of the ban’s effectiveness in reducing minors’ access to EDs (*p* = 0.81).

Place of residence was not significantly associated with support for the ban (H(4, *n* = 871) = 4.16; *p* = 0.385; χ^2^ = 0.00; *p* = 1.000) or with perceptions of its effectiveness (H(4, *n* = 871) = 7.74; *p* = 0.102; χ^2^ = 4.88; *p* = 0.300). Nonetheless, pairwise comparisons indicated that students from rural areas were more supportive of additional restrictions compared to those from small towns (*p* = 0.017) and large cities (*p* = 0.039).

Other sociodemographic variables, including year and type of study program, financial situation, and study mode (full-time vs. part-time), were not significantly associated with attitudes toward EDs regulations. The comparison of attitudes by gender is shown in Table 2.

## 4. Discussion

The findings indicate that medical students demonstrated higher awareness of ED ingredients and potential adverse effects than non-medical students, particularly with respect to caffeine, simple sugars, B vitamins, L-carnitine, and electrolytes, while knowledge about taurine, guarana, and ginseng remained similar across groups. It has been reported that while students generally acknowledge caffeine and sugar in EDs, their understanding of additional ingredients such as amino acids and B vitamins remains limited, including among those in medical fields [11,19].

A study among university students in Jordan showed that higher knowledge scores were associated with studying a medical major [13]. Likewise, evidence from Italy indicated that students of life sciences courses were more likely to be ED users and had greater awareness of their ingredients and related behaviors [9]. These results suggest that the field of study plays an important role in shaping the awareness of ED contents.

Analysis of consumption behaviors did not reveal significant differences between medical and non-medical students in terms of the frequency of ED use, nor in the co-consumption of these products with alcohol, cigarettes, or other psychoactive substances. The only distinction observed was a higher tendency among medical students to combine EDs with coffee, while the reported age of initiation was similar across groups. Comparable findings were reported in a Saudi study, where gender and field of study emerged as determinants of ED consumption, although not all behavioral aspects differed consistently between groups [11]. Likewise, research among Jordanian students has shown that although knowledge about EDs varies by academic discipline, actual consumption practices often remain similar, with motives such as staying awake or enhancing study performance prevailing across groups [13]. These results suggest that while medical education may increase awareness of potential risks, it does not necessarily translate into markedly different consumption patterns when compared to non-medical peers.

In the present study, medical students were more likely to indicate energy and concentration as primary motives for consuming EDs, whereas leisure and socially driven motives did not differ significantly between groups, suggesting similar behavioral patterns in this domain. Similar patterns have been observed in other research, where study-related demands were identified as a predominant reason for ED consumption among medical students [7].

Among the analyzed sociodemographic variables, gender emerged as the strongest factor differentiating attitudes toward regulation, whereas other factors such as year and mode of study or financial situation were not of significant importance. Similar findings were reported in previous studies, where gender emerged as a key determinant of ED use and related attitudes, while other sociodemographic variables such as academic year or financial status showed weaker associations [20,21,22,23,24,25].

A high level of support for the ban on the sale of EDs to individuals under the age of 18 was observed in both groups. However, sociodemographic analysis revealed gender differences. Female students were more likely than males to support the ban as well as additional restrictions, such as advertising limitations. With regard to the perceived effectiveness of the ban, no significant differences were noted by gender or place of residence. Nevertheless, a more detailed analysis indicated that students from rural areas expressed stronger support for additional restrictions compared to those living in small and large cities. Similar gender-based differences were reported in a recent nationally representative study of adults in Poland, where women were significantly more likely than men to perceive EDs as harmful to health (85.6% vs. 80.1%; *p* = 0.02), to support the sales ban to minors (89.5% vs. 84.4%; *p* = 0.01), and to endorse additional restrictions such as advertising bans (74.4% vs. 61.9%; *p* < 0.001). These findings are consistent with the results of the present study, suggesting that gender-related differences in attitudes towards EDs regulation extend beyond student populations and may reflect broader societal patterns [4].

The observed patterns of ED use among future health professionals highlight the importance of addressing potentially unhealthy consumption habits early in training and integrating targeted health education to promote safer behaviors [26]. Given the evidence linking ED use among nurses to poorer sleep quality, reduced sleep duration, and higher stress levels, it is particularly important to monitor and address ED consumption among medical students, as their future professional roles in demanding clinical environments may predispose them to increased intake of these beverages [27,28,29]. Moreover, greater attention from policymakers is needed to strengthen regulations on the advertising of EDs and other high-caffeine beverages, to which adolescents and young adults including future healthcare providers are widely exposed. Increasing knowledge about EDs and their possible risks could also decrease their consumption by the general public, further supporting preventive efforts [30,31]. Other research has also shown that medical students tend to demonstrate better knowledge of energy drink ingredients compared with non-medical students, yet this awareness does not necessarily translate into lower consumption [32,33]. Awareness campaigns and educational programs targeting both medical and non-medical students could help improve understanding of energy drink ingredients, associated risks, and safe consumption practices [34]. Increasing knowledge about how excessive ED use can affect concentration, sleep, and overall well-being may help promote healthier study habits and reduce reliance on stimulants during academic activities [35]. The findings of this study emphasize the importance of addressing energy drink use within medical education. As future healthcare professionals, medical students will be responsible for counseling patients on healthy lifestyle behaviors, including the risks of caffeine-containing beverages.

### Limitations

Given the cross-sectional design, findings reflect associations and do not allow causal inference. The use of self-reported data may introduce recall bias and social desirability bias. Moreover, convenience sampling via social media may limit generalizability, as students more engaged online could be overrepresented. These limitations were partially mitigated through pilot testing of the tool, reliability checks, wide distribution across diverse study groups, and an adequately powered sample size. In addition, the presented group may not fully reflect the structure of the academic population in Poland. Women were overrepresented in the sample (approximately 74%) compared to national statistics, where they constitute around 58% of students according to Statistics Poland (GUS) [36]. This imbalance may partly reflect a greater willingness of female students to participate in surveys.

## 5. Conclusions

This study shows that although medical students are more aware of ED ingredients and risks, their consumption patterns remain similar to non-medical students. Energy and concentration remain the main reasons for use, while social motives are less common. Students widely support the ban on sales to minors but doubt its effective enforcement; gender differences most strongly shape regulatory attitudes. These findings highlight the need to complement legislation with targeted health education, especially within medical curricula, as future shift-based work may increase stimulant use, and with effective monitoring to strengthen policy impact and reduce risky consumption among young adults.

## Figures and Tables

**Table 1:** Characteristics of the study group.

Category	M	SD	Min–Max
**Age**	22.12	3.05	18–45
		***n***	**%**
**Sex**	Female	638	73.25
	Male	220	25.26
	Other	7	0.80
	Prefer not to disclose	6	0.69
**Place of residence**	Countryside (village)	89	10.22
	Small town (up to 20,000)	47	5.40
	Medium-sized city (20,000–100,000)	95	10.91
	Large city (100,000–500,000)	58	6.66
	Metropolitan city (>500,000)	582	66.82
**Financial situation**	Very good	250	28.70
	Good	517	59.36
	Difficult to say	86	9.87
	Poor	16	1.84
	Very poor	2	0.23
**Type of study program**	Bachelor’s degree	328	37.66
	Master’s degree	185	21.24
	Integrated master’s program	358	41.10
**Mode of study**	Full-time	743	85.30
	Part-time	128	14.70
**Year of study**	I	201	23.08
	II	227	26.06
	III	200	22.96
	IV	136	15.6
	V	82	9.41
	VI	25	2.87
**Filed of study**	Medical	367	42.14
	Non-medical	504	57.86

Note: Data are presented as mean (M), standard deviation (SD), and range (Min–Max) for continuous variables, and as number (*n*) and percentage (%) for categorical variables. M, mean; SD, standard deviation. This table presents descriptive data only; no inferential statistical tests were applied.

**Table 2:** Comparison of attitudes toward ED regulations between female and male students.

Variable	Sum of Ranks (Female)	Sum of Ranks (Male)	U	Z	*p*	Z (Continuity-Corrected)	*p* (Continuity-Corrected)	*n* (Female)	*n* (Male)
Ban on the sale of EDs to people under 18 is justified	291,057.5	77,453.5	53,143.5	13,636	<0.001	45,906	<0.001	638	220
The introduction of a ban on the sale of EDs to minors in Poland has effectively reduced their availability to underage individuals	273,268.5	95,242.5	69,427.5	−0.24	0.812	−0.25	0.806	638	220
Additional restrictions on EDs, such as advertising limitations, should be implemented	296,763.5	71,747.5	47,437.5	42,917	<0.001	14,793	<0.001	638	220

Note: Statistical analyses were performed using the Mann–Whitney U test (two-tailed) with continuity correction. Significant results are indicated at *p* < 0.05. Data are shown as sum of ranks, U statistic, Z value, and *p*-value for each comparison. EDs, energy drinks.

## Data Availability

The data supporting the results of this study will be available upon request from interested researchers. If the data cannot be made publicly available in a trusted repository, the reason for this will be specified in the Data Availability Statement. Further information and materials necessary for the reproduction of the experiment can be obtained by contacting the authors.
